# Use of fibrates is not associated with reduced risks of mortality or cardiovascular events among ESRD patients: A national cohort study

**DOI:** 10.3389/fcvm.2022.907539

**Published:** 2022-11-09

**Authors:** Wen-Yu Ho, Chieh-Li Yen, Cheng-Chia Lee, Yi-Ran Tu, Chao-Yu Chen, Ching-Chung Hsiao, Pao-Hsien Chu, Hsiang-Hao Hsu, Ya-Chun Tian, Chih-Hsiang Chang

**Affiliations:** ^1^Department of Nephrology, Chang Gung Memorial Hospital and College of Medicine, Chang Gung University, Taoyuan, Taiwan; ^2^Kidney Research Center, Chang Gung Memorial Hospital, Taoyuan, Taiwan; ^3^Department of Cardiology, Chang Gung Memorial Hospital and College of Medicine, Chang Gung University, Taoyuan, Taiwan; ^4^Institute of Stem Cell and Translational Cancer Research, Chang Gung Memorial Hospital, Taoyuan, Taiwan

**Keywords:** fibrates, hypertriglyceridemia, end-stage renal disease, cardiovascular, mortality

## Abstract

**Background:**

Although a recent study reported that fibrates are associated with a low risk of cardiovascular (CV) death and can postpone the need for long-term hemodialysis in patients with advanced chronic kidney disease (CKD), little is known regarding whether the CV protective effects of fibrates extend to patients with end-stage renal disease (ESRD). The present study compared CV outcomes and mortality among patients with ESRD treated with fibrates, statins, neither, or their combination.

**Methods:**

This cohort study extracted data from Taiwan's National Health Insurance Research Database (NHIRD). Adult patients with ESRD and hyperlipidemia were identified and categorized into four groups (fibrate, statin, combination, and non-user groups) according to their use of different lipid-lowering therapies within 3 months prior to the commencement of permanent dialysis. Inverse probability of treatment weighting was used to balance the baseline characteristics of the groups. The follow-up outcomes were all-cause mortality, CV death, and major adverse cardiac and cerebrovascular events (MACCEs).

**Results:**

Compared with the non-user and statin groups, the fibrate group did not exhibit significantly lower risks of all-cause mortality [fibrate vs. non-user: hazard ratio (HR), 0.97; 95% confidence interval (CI), 0.92–1.03; statin vs. fibrate: HR, 0.95; 95% CI, 0.90–1.01], CV death (fibrate vs. non-user: HR, 0.97; 95% CI, 0.90–1.05; statin vs. fibrate: HR, 0.97; 95% CI, 0.90–1.06), and MACCEs (fibrate vs. non-user: HR, 1.03; 95% CI, 0.96–1.10; statin vs. fibrate: HR, 0.94; 95% CI, 0.87–1.004). The combination of fibrates and statins (specifically moderate- to high-potency statins) did not result in lower risks of all-cause mortality, CV death, or MACCEs compared with statins alone.

**Conclusion:**

In patients with ESRD, the use of fibrates might be not associated with reduced mortality or CV risks, regardless of whether they are used alone or in combination with statins.

## Introduction

Cardiovascular disease (CVD) is the leading cause of death in patients with end-stage renal disease (ESRD) and accounts for 40–50% of mortality among such patients (1). The risk of CVD increases with the decline of kidney function (2). In the general population, atherosclerosis is the primary cause of CVD. Common risk factors for atherosclerotic cardiovascular disease (ASCVD) have been identified, and several preventive strategies have been demonstrated to effectively reduce the risk of ASCVD. One of the most effective preventive measures is lipid-lowering therapy, which mainly consists of two categories of medications: statins and fibrates. Although statins are considered the most powerful lipid-lower agents reducing ASCVD risk, they seem to be less effective for patients with chronic kidney disease (CKD) and ESRD than for the general population (3). This discrepancy may be attributed to alterations in the lipid metabolism pathway that accompany CKD progression. Compared with statins, the reduction of triglyceride (TG) levels by fibrates is thought to play a minor role in cardiovascular protection in the general population; however, studies have revealed that TG-rich lipoproteins are causal risk factors for ASCVD (4–7). Medications that decrease TG or TG-rich lipoproteins reduce the risk of CVD in patients with hypertriglyceridemia, especially in those with low levels of high-density lipoprotein (HDL) (8, 9). Hypertriglyceridemia is a hallmark lipid abnormality in patients with CKD (10, 11) and mainly results from the dysfunction of lipoprotein lipase (LPL) and hepatic lipase that are responsible for the degradation of TG-rich chylomicron and low-density lipoprotein (VLDL) (11, 12). Decreased levels and functional disturbance of HDL, which are caused by low levels of apoprotein (Apo) A-I, ApoA-II, and lecithin-cholesterol acyltransferase (LCAT) (12, 13), are other lipid abnormalities commonly observed in patients with CKD. Because they can simultaneously decrease TG and elevate HDL levels (14), fibrates might be more beneficial for patients with CKD than for the general population.

Fibrates are activators of the nuclear transcription receptor peroxisome proliferator–activated receptor (PPAR)-α, which modulates the synthesis of multiple proteins involved in lipid metabolism. Fibrates upregulate the expression of LPL and downregulate the expression of ApoC-III, an LPL inhibitor, thus reduce the levels of TG and TG-rich lipoproteins in the blood. Meanwhile, fibrates upregulate ApoA-I and ApoA-II, thereby increasing HDL levels (15). Fibrates are primarily eliminated through urine and have prolonged half-lives and elevated concentrations in patients with renal insufficiency. This holds true for patients undergoing dialysis because fibrate metabolites are non-dialyzable from the serum (16). Because of such concerns, the use of fibrates for the treatment of dyslipidemia in CKD population remains limited. Studies have evaluated the effectiveness of fibrates in reducing CV risk in populations with mild to moderate (14, 17, 18) and advanced (19) CKD. However, few studies have evaluated the effectiveness of fibrates in reducing CV risk in patients with ESRD. A randomized controlled single-center study demonstrated that fibrates could effectively reduce lipid levels and oxidative stress in patients with ESRD (20). However, whether fibrates effectively reduce CV risk in patients with ESRD, as they do in patients with CKD, remains unknown. We conducted a well-designed, high-quality, large-scale, population-based cohort study to investigate this question. We used data from Taiwan's National Health Institute Research Database (NHIRD) to compare the effects of fibrates and statins, used separately and concurrently, on CV outcomes and mortality among patients with ESRD.

## Materials and methods

### Data source

This retrospective cohort study collected data from Taiwan's NHIRD. In 1995, the Taiwanese government established the National Health Insurance (NHI) program, a single-payer, mandatory insurance system in which most health-care facilities are enrolled. By the end of 2014, more than 99.9% of Taiwan's population was covered by the NHI program. Physicians are required to upload claims data from each outpatient or inpatient visit. The NHIRD was established by Taiwan's National Health Research Institutes in 2002 for public research purposes, and the cohort of this database is one of the largest health-care cohorts in the world. The NHIRD provides detailed health-care information, including basic demographic information, disease diagnoses, medicine prescriptions, procedural interventions, inpatient management information, and registrations of special conditions, but laboratory data and examination reports are not included in this database. Disease diagnoses in the NHIRD records are made according to the *International Classification of Diseases, Ninth Edition, Clinical Modification* (*ICD-9-CM*). More details on the NHIRD and methodologic approaches for data validation are provided in previous studies (21, 22). This study was approved with a waiver of consent by the Institutional Review Board of Chang Gung Medical Foundation (Approval Number: 201900840B0).

### Study design

**Figure 1:**
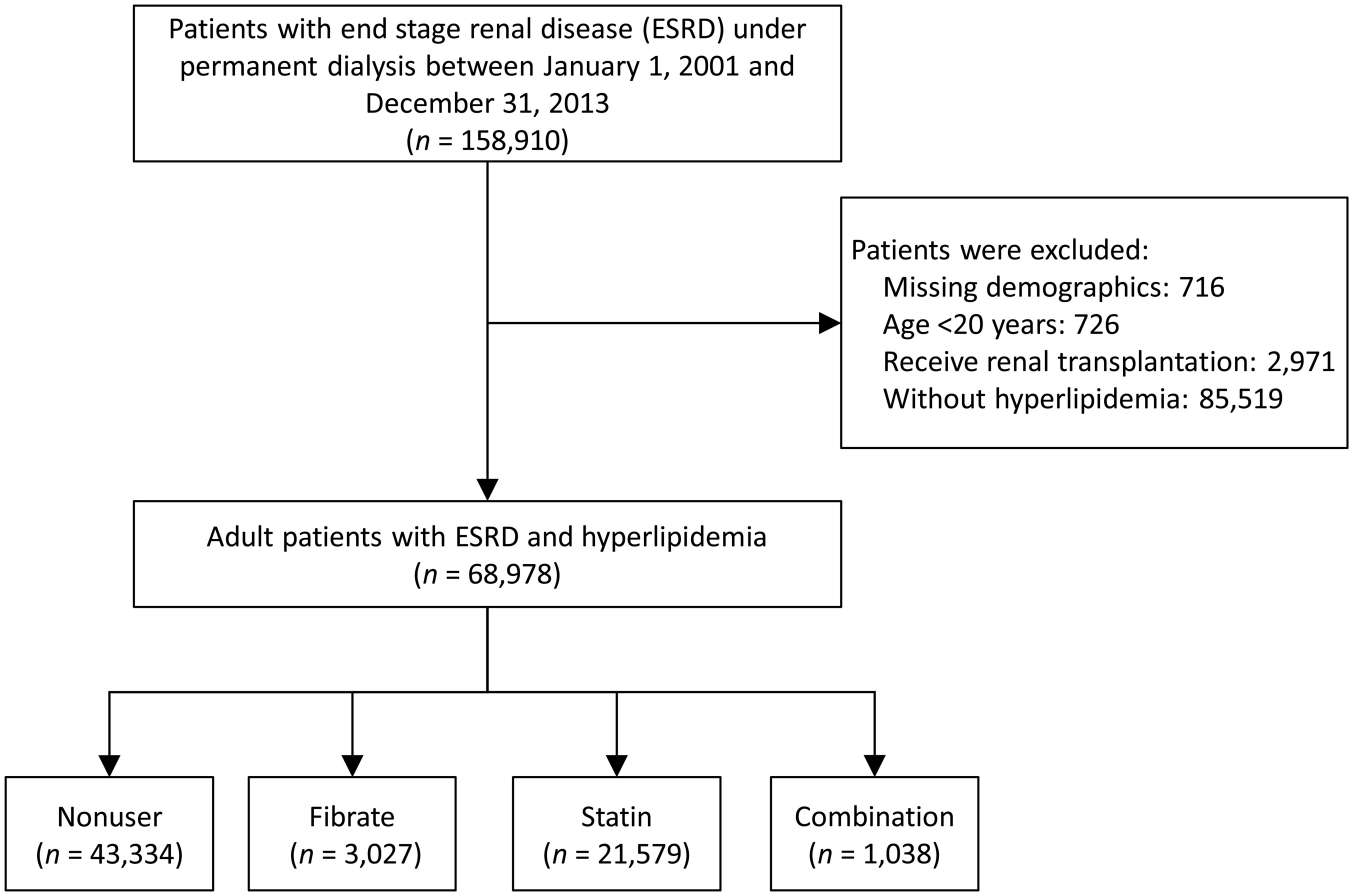
Flowchart for illustrating the inclusion and exclusion of the patients.

We collected data from the NHIRD to determine the effects of fibrates and statins on patients with ESRD and compare the study outcomes of patients using fibrates, statins, neither, or their combination. As shown in Figure 1, we identified patients with ESRD and concurrent hyperlipidemia who underwent permanent dialysis between January 1, 2001, and December 31, 2013. A patient was identified as having ESRD if they had a catastrophic illness certificate for long-term hemodialysis or peritoneal dialysis. The strict review process of the catastrophic illness certificate in Taiwan made the diagnosis of ESRD reliable; the index date was defined as the date on which they obtained the certification.

Patients without previous diagnosis of any kind of hyperlipidemia, with incomplete demographics, who were younger than 20 years, or who had undergone renal transplantation prior to the index date were excluded. The remaining patients were divided into four groups (fibrate, statin, combination, and non-user groups) according to their use of lipid-lowering medications within 3 months prior to the index date (19). In Taiwan, cholestyramine is not available, and, according to NHI's regulations, ezetimibe and niacin could only be prescribed with statin for patients who are difficult to achieve treatment target under statin alone. Thus, about the lipid-lowering medications, we only considered the use of fibrate and statin in this study. Besides, the drug exposure period (within 3 months prior to the index date) was according to the definition of “current use of medication” in a previous study (23). The follow-up period started on the index date and ended on the date of occurrence of any study outcomes, the date of withdrawal from the Taiwan NHI system (usually death), or the end of the study period (December 31, 2013), whichever occurred first. The Taiwan NHI is a single insurance system and data are centralized, therefore the lost to follow up of the insured individuals is rare and the threat of attrition bias is low.

### Covariates

The covariates in the study were age, gender, CKD duration, number of outpatient department visits in the year prior to the index date, comorbidities (hypertension, diabetes mellitus, atrial fibrillation, liver cirrhosis, peripheral artery disease, dementia and immune disease), history of events (hospitalization for heart failure, stroke, and myocardial infarction), and twenty kinds of medication. The CKD duration was defined as the time between the date of CKD diagnosis (first record of CKD codes) and the index date. Comorbidities were defined as diseases that were reported within 1 year of the index date and required hospitalization or more than two outpatient follow-ups. The history of events (heart failure, stroke, and myocardial infarction) was defined by the occurrence of event-associated hospitalizations prior to the index date. Medications were determined using both outpatient and inpatient prescription records from the 3 months prior to the index date. The definition of comorbidities and identification of medications in this study were adopted by many previous high-quality NHIRD-based studies (19, 24, 25). The moderate-to-high-potency statins indicated the statins could reduce LDL-C levels by more than 30% (26).

### Outcomes

The study outcomes of interest were all-cause mortality, CV death, and major adverse cardiac and cerebrovascular events (MACCEs). All-cause mortality was detected based on a withdrawal from the Taiwan NHI program (27). In Taiwan, the main reason for withdrawal from the NHI program is death. The other less common reasons are permanent emigration, missing >6 months or being jailed for more than 6 months. CV deaths were defined as deaths resulting from acute myocardial infarction, sudden cardiac death, heart failure, stroke, CV procedures, CV hemorrhage, or other CV causes (28). MACCEs, namely cardiovascular death, ischemic stroke, and acute myocardial infarction, were determined according to the main discharge diagnoses of hospitalizations or ER visits. The diagnostic codes of acute myocardial infarction (29) and ischemic stroke (30, 31) have been validated in previous NHIRD studies.

### Statistical analysis

The baseline characteristics of patients with different lipid lowering therapies (fibrate, statin, combination, and non-user groups) were balanced using inverse probability of treatment weighting (IPTW) based on the generalized propensity score of multiple treatments (32). The propensity scores were generated using the generalized boosted model (GBM) based on 50,000 regression trees. Compared to the conventional methods (i.e., multinomial logistic regression model), the GBM method has shown superior performance in most of the scenarios (33). Covariates used to calculate the propensity scores were all of the variables listed in Table 1, where the follow up duration was replaced with the index date. The balance among the multiple treatment groups before and after IPTW was assessed using the maximum absolute standardized difference (MASD) between any two of the groups, where a value <0.1 (34) indicated negligible difference between groups.

**Table 1:** Baseline characteristics of dialytic patients according to the use of fibrate and statin before IPTW adjustment.

**Variable**	**Non-user (*n* = 43,334)**	**Fibrate (*n* = 3,027)**	**Statin (*n* = 21,579)**	**Combination (*n* = 1,038)**	**MASD**
Age, year	65.7 ± 12.7	61.1 ± 12.3	62.6 ± 12.4	59.7 ± 11.1	0.47
**Age group**					0.50
20–64 years	19,387 (44.7)	1,824 (60.3)	12,054 (55.9)	709 (68.3)	
65–74 years	12,742 (29.4)	785 (25.9)	5,918 (27.4)	229 (22.1)	
≥75 years	11,205 (25.9)	418 (13.8)	3,607 (16.7)	100 (9.6)	
Male	21,171 (48.9)	1,461 (48.3)	10,155 (47.1)	445 (42.9)	0.12
CKD duration, year	5 [3, 8]	4 [3, 7]	4 [2, 8]	4 [3, 7]	0.13
No. of outpatient visit in the previous year	8 [1, 17]	7 [1, 15]	10 [3, 17]	8 [1, 15]	0.21
**Comorbid conditions**					
Hypertension	38,856 (89.7)	2,514 (83.1)	19,995 (92.7)	906 (87.3)	0.32
Diabetes mellitus	30,744 (70.9)	2,187 (72.2)	17,401 (80.6)	824 (79.4)	0.22
Atrial fibrillation	1,697 (3.9)	86 (2.8)	632 (2.9)	15 (1.4)	0.13
Liver cirrhosis	1,562 (3.6)	61 (2.0)	528 (2.4)	14 (1.3)	0.13
Peripheral artery disease	2,066 (4.8)	136 (4.5)	1,005 (4.7)	51 (4.9)	0.02
Dementia	1,892 (4.4)	78 (2.6)	574 (2.7)	24 (2.3)	0.11
Immune disease	1,010 (2.3)	59 (1.9)	448 (2.1)	21 (2.0)	0.03
**History of event**					
Heart failure	13,667 (31.5)	724 (23.9)	6,651 (30.8)	269 (25.9)	0.17
Stroke	10,813 (25.0)	642 (21.2)	5,023 (23.3)	221 (21.3)	0.09
Myocardial infarction	3,949 (9.1)	227 (7.5)	2,653 (12.3)	119 (11.5)	0.16
**Medication**					
ACEi/ARB	19,573 (45.2)	1,457 (48.1)	12,588 (58.3)	588 (56.6)	0.26
Beta blocker	20,680 (47.7)	1,602 (52.9)	12,961 (60.1)	639 (61.6)	0.28
DCCB	28,856 (66.6)	1,955 (64.6)	16,732 (77.5)	712 (68.6)	0.28
Loops diuretics	24,119 (55.7)	1,511 (49.9)	15,087 (69.9)	627 (60.4)	0.41
Spironolactone	1,048 (2.4)	50 (1.7)	681 (3.2)	30 (2.9)	0.09
NDCCB	3,330 (7.7)	255 (8.4)	2,145 (9.9)	105 (10.1)	0.09
Oral hypoglycemic agents	16,209 (37.4)	1,304 (43.1)	10,791 (50.0)	486 (46.8)	0.26
Insulin	10,967 (25.3)	1,121 (37.0)	8,300 (38.5)	508 (48.9)	0.51
Antiplatelet	13,324 (30.7)	1,109 (36.6)	9,797 (45.4)	470 (45.3)	0.31
Oral anticoagulants	1,114 (2.6)	90 (3.0)	569 (2.6)	35 (3.4)	0.05
NSAIDs	6,456 (14.9)	604 (20.0)	2,985 (13.8)	204 (19.7)	0.17
Steroid	3,540 (8.2)	222 (7.3)	1,819 (8.4)	70 (6.7)	0.06
Proton pump inhibitor	7,240 (16.7)	526 (17.4)	3,735 (17.3)	177 (17.1)	0.02
Ketosteril	1,419 (3.3)	50 (1.7)	787 (3.6)	22 (2.1)	0.11
Pentoxifylline	5,190 (12.0)	389 (12.9)	3,788 (17.6)	153 (14.7)	0.16
Sodium bicarbonate	3,465 (8.0)	157 (5.2)	1,918 (8.9)	64 (6.2)	0.14
Immunosuppressants	600 (1.4)	31 (1.0)	350 (1.6)	20 (1.9)	0.08
Vitamin D	3,480 (8.0)	241 (8.0)	1,914 (8.9)	95 (9.2)	0.04
Iron supplement	6,352 (14.7)	396 (13.1)	3,739 (17.3)	147 (14.2)	0.12
Calcium	12,447 (28.7)	961 (31.7)	6,877 (31.9)	342 (32.9)	0.09
Follow-up year	3.2 ± 3.0	4.2 ± 3.5	3.3 ± 2.9	4.1 ± 3.5	0.51

IPTW, inverse probability of treatment weighting; CKD, chronic kidney disease; MASD, maximum absolute standardized difference; ACEi, angiotensin converting enzyme inhibitor; ARB, angiotensin receptor blocker; DCCB, dihydropyrinde calcium channel blocker; NDCCB, non-dihydropyrinde calcium channel blocker; NSAIDs, non-steroidal anti-inflammatory drugs; Data were presented as frequency (percentage), median [25th, 75th percentile] or mean ± standard deviation.

The risk of the study outcomes in patients with different lipid lowering therapies was compared using the Cox proportional hazard model. The study group (fibrate, statin, combination, and non-user) was the only explanatory variable in the Cox model. All comparisons between any two groups were made and a total of six pairwise comparisons were obtained for each outcome. Furthermore, the usage of statins was restricted on moderate to high potency statins and the propensity scores as well as GBM-IPTW were re-calculated. A two-sided *P*-value <0.05 was considered to be statistically significant. All the statistical analyses were performed using SAS version 9.4 (SAS Institute, Cary, NC).

## Results

### Patient characteristics

As shown in Figure 1, the data of 68,978 patients with ESRD and hyperlipidemia diagnosed between January 1, 2001, and December 31, 2013, were extracted from the entire Taiwanese population. Of these patients, 3,027 used fibrates (fibrate group), 21,579 used statins (statin group), 1,038 used a combination of fibrates and statins (combination group), and the remaining 43,334 had not used any type of lipid-lowing agent (non-user group) within the 3 months prior to the index date.

The baseline characteristics, namely demographics, comorbidities, history of certain events, and prescribed medications, of the groups are presented in Table 1. Before inverse probability of treatment weighting (IPTW) was applied, the statin and combination groups—compared with the fibrate and non-user groups—were generally younger and had more OPD visits; a higher prevalence of hypertension and diabetes mellitus; and higher proportions of patients using certain medications, namely angiotensin-converting enzyme inhibitors/angiotensin receptor blockers, dihydropyridine calcium channel blockers, loop diuretics, oral hypoglycemic agents, insulin, and antiplatelets. After IPTW, all the MASD values were ≤ 0.1, which indicated that the baseline characteristics and follow-up durations of all the groups were well-balanced (Supplementary Table 1).

### Follow-up outcomes

**Figure 2:**
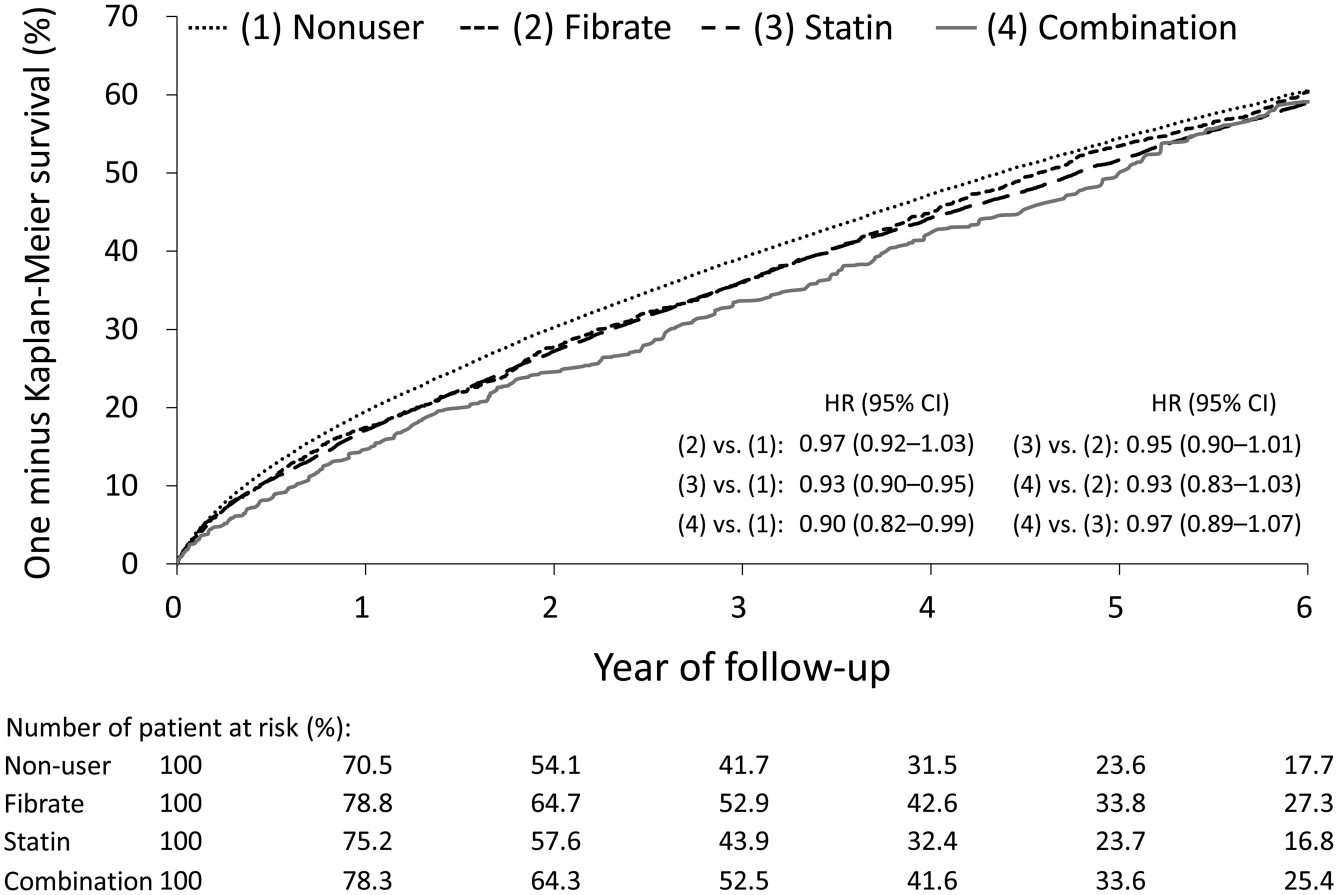
One minus Kaplan-Meier survival curves of the patients with concomitant end stage renal disease and hyperlipidemia under different lipid lowering therapies in the IPTW-adjusted cohort. IPTW, inverse probability of treatment weighting.

The follow-up outcomes of the study groups after IPTW are listed in Table 2. The statin and combination groups exhibited a significantly lower risk of all-cause mortality compared with the non-user group; however, compared with the non-user and statin groups, the fibrate group did not exhibit significantly lower risks of all-cause mortality [fibrate vs. non-user: hazard ratio (HR), 0.97; 95% confidence interval (CI), 0.92–1.03; statin vs. fibrate: HR, 0.95; 95% CI, 0.90–1.01], CV death (fibrate vs. non-user: HR, 0.97; 95% CI, 0.90–1.05; statin vs. fibrate: HR, 0.97; 95% CI: 0.90–1.06), and MACCEs (fibrate vs. non-user: HR, 1.03; 95% CI, 0.96–1.10; statin vs. fibrate: HR, 0.94; 95% CI, 0.87–1.004). Similarly, between the combination and statin groups, no significant differences were identified in all-cause mortality (HR, 0.97; 95% CI, 0.89–1.07), CV death (HR, 1.02; 95% CI, 0.89–1.17), or MACCEs (HR, 1.04; 95% CI, 0.93–1.17). The one-minus-Kaplan–Meier survival rates of the four groups are presented in Figure 2.

**Table 2:** Follow up outcome for the dialytic patients according to the use of fibrate and statin in the IPTW-adjusted cohort.

	**Incidence^*$*^**	**HR (95% CI) (Column vs. row)**		
**Outcome/group**	**(95% CI)**	**Fibrate**	**Statin**	**Combination**
**All-cause mortality**				
Non-user	16.6 (16.4–16.7)	0.97 (0.92–1.03)	0.93 (0.90–0.95)*	0.90 (0.82–0.99)*
Fibrate	16.0 (15.8–16.2)	–	0.95 (0.90–1.01)	0.93 (0.83–1.03)
Statin	15.3 (15.1–15.4)	–	–	0.97 (0.89–1.07)
Combination	14.9 (14.7–15.0)	–	–	–
**Cardiovascular death**				
Non-user	8.7 (8.6–8.8)	0.97 (0.90–1.05)	0.94 (0.91–0.98)*	0.96 (0.84–1.10)
Fibrate	8.4 (8.3–8.5)	–	0.97 (0.90–1.06)	0.99 (0.85–1.15)
Statin	8.2 (8.0–8.3)	–	–	1.02 (0.89–1.17)
Combination	8.3 (8.2–8.4)	–	–	–
**MACCE** ^**#**^				
Non-user	12.3 (12.2–12.5)	1.03 (0.96–1.10)	0.96 (0.94–0.99)*	1.00 (0.89–1.13)
Fibrate	12.7 (12.5–12.8)	–	0.94 (0.87–1.004)	0.97 (0.85–1.11)
Statin	11.8 (11.7–12.0)	–	–	1.04 (0.93–1.17)
Combination	12.3 (12.1–12.4)	–	–	–

IPTW, inverse probability of treatment weighting; HR, hazard ratio; CI, confidence interval; MACCE, major adverse cardiac and cerebrovascular events; ^*$*^Incidence density was presented as event numbers per 100 person-years; ^#^Composite of cardiovascular death, ischemic stroke, or acute myocardial infarction; ^*^P < 0.05. Bold values denote statistical significance at the P < 0.05.

Because the duration of the use of lipid-lowering agents may affect the results. We selected patients who initiated statin, fibrate, or combination treatment since 3 months before index date and were still under treatment within 3 months prior to index date for analysis. The main results across long-term fibrate group, long-term statin group, long-term combination group were still consistent (Supplementary Table 4).

### Follow-up outcomes for fibrates and moderate- to high-potency statins

A previous study reported that the combination of high-potency statins and fibrates has potential benefits for patients with advanced CKD (19). In this study, we determined whether this combination might benefit patients with ESRD. We excluded patients using low-potency statins and reperformed IPTW to rebalance the groups (Supplementary Tables 2, 3 present the baseline information of the modified groups); the follow-up outcomes are presented in Table 3. The moderate- to high-potency statin group exhibited modest declines in all-cause mortality, CV death, and MACCEs relative to the non-user group, and modest declines in MACCEs relative to the fibrate group; however, no significant differences were identified between the combination group and the moderate- to high-potency statin or fibrate groups.

**Table 3:** Follow up outcomes for the dialytic patients according to the use of fibrate and moderate- to high-potency statins in the IPTW-adjusted cohort.

	**Incidence^*$*^**	**HR (95% CI) (Column vs. row)**		
**Outcome/group**	**(95% CI)**	**Fibrate**	**Moderate- to high-potency statins**	**Combination**
**All-cause mortality**				
Non-user	16.7 (16.5–16.9)	0.97 (0.91–1.02)	0.91 (0.89–0.94)*	0.88 (0.79–0.98)*
Fibrate	16.0 (15.9–16.2)	–	0.95 (0.89–1.004)	0.91 (0.81–1.03)
Moderate- to high-potency statins	15.2 (15.0–15.4)	–	–	0.96 (0.86–1.08)
Combination	14.6 (14.5–14.8)	–	–	–
**Cardiovascular death**				
Non-user	8.8 (8.6–8.9)	0.97 (0.89–1.05)	0.93 (0.90–0.97)*	0.97 (0.83–1.13)
Fibrate	8.4 (8.3–8.6)	–	0.96 (0.89–1.05)	1.00 (0.85–1.19)
Moderate- to high-potency statins	8.1 (8.0–8.2)	–	–	1.04 (0.89–1.21)
Combination	8.4 (8.3–8.6)	–	–	–
**MACCE** ^**#**^				
Non-user	12.4 (12.3–12.6)	1.03 (0.96–1.10)	0.96 (0.92–0.99)*	1.01 (0.89–1.15)
Fibrate	12.7 (12.6–12.9)	–	0.93 (0.87–0.997)*	0.98 (0.85–1.13)
Moderate- to high-potency statins	11.8 (11.7–12.0)	–	–	1.06 (0.93–1.21)
Combination	12.4 (12.3–12.6)	–	–	–

IPTW, inverse probability of treatment weighting; HR, hazard ratio; CI, confidence interval; MACCE, major adverse cardiac and cerebrovascular events; ^*$*^Incidence density was presented as event numbers per 100 person-years; ^#^Composite of cardiovascular death, ischemic stroke, or acute myocardial infarction; ^*^ *P* < 0.05. Bold values denote statistical significance at the P < 0.05.

## Discussion

The role of fibrates in the reduction of mortality or CV risk among patients with CKD or ESRD have yet to be thoroughly studied. Although hypertriglyceridemia is commonly observed among patients undergoing permanent dialysis, nephrologists have difficulty in deciding whether to treat it with fibrates because of the lack of relevant researches. We designed this nationwide cohort study to compare all-cause mortality, CV deaths, and MACCEs among patients with ESRD using fibrates, statins, neither, or both to determine whether fibrates can reduce the risks of mortality and CV events in these patients.

Fibrates, a second-line lipid-lowering therapy, are not commonly used in patients with kidney impairment because they are mostly eliminated through urine and their metabolites might accumulate in patients with advanced kidney disease (16). A randomized controlled study reported that long-term fenofibrate (200 mg per day) use was associated with a reduced risk of CV events in patients with moderate kidney impairment (eGFR: 30–59 mL/min/1.73 m^2^) (17). Our research team conducted a population-based cohort study, revealing that fibrates can postpone the necessity of permanent dialysis and can reduce the risk of CV death in patients with advanced CKD. In subgroup analysis, the previous study also reported that the combination of fibrates and high-intensity statins exerted a stronger protective effect against CV events, though this relationship was less evident because of the fewer patients in the subgroup analyses. Furthermore, reduced-dose, alternate-day administration of fibrates can be safely used to treat patients undergoing hemodialysis, and fibrates exert antioxidative effects in addition to lipid-lowering effects, with the only side effect being a non-significant elevation of muscle enzymes (20, 35). However, in the present study, patients who used fibrates did not exhibit outcomes (i.e., all-cause mortality, CV death, or MACCEs) superior to those of patients who used statins or who did not use lipid-lowering agents. Furthermore, the combination of statins and fibrates exhibited no additional benefits beyond those exhibited by statins or fibrates alone, regardless of whether the statins used had moderate-to-high potency. By comparison, this study exhibited the modest reduction in the study outcomes in the statin group. Although previous 4D and AURORA trials indicated that, compared with the impressive CV protection of statin treatment in non-ESRD population, statins have much less benefits for patients with ESRD (36, 37). However, this study demonstrated that statins, especially the moderate-to-high potency statins, still have better performances in reducing CV events than fibrates do among new-onset ESRD patients.

The possible explanations of why the TG-lowering and antioxidative properties of fibrates did not translate into reductions in the rates of the study outcomes are discussed as follows. First, the most common cause of mortality among patients with ESRD is sudden cardiac death, which accounts for ~50% of such mortalities, followed by non-sudden CV disease and non-cardiac causes; these causes differ considerably from in the main causes of mortality among patients with CKD not undergoing dialysis (38), of which the most common cause is ASCVD. Therefore, reductions in traditional risk factors for ASCVD may not strongly affect overall mortality among patients with ESRD. Second, hemodialysis is associated with additional CV risks (39), namely sudden changes in blood pressure, use of anticoagulants, arteriosclerosis induced by calcium–phosphate imbalance, and frequent blood loss during hemodialysis. Therefore, hemodialysis considerably affects CV outcomes. Third, the chronic inflammation among patients with ESRD, including decreased clearance of pro-inflammatory cytokines, recurrent infections, and intestinal dysbiosis, have been proved to increase the CV risks (40), which is less influenced by TG-lowering agents. In patients with ESRD, factors not affected by TG-lowering therapies might dominate the causal pathway of adverse CV outcomes and thus obscure the effect of these drugs.

Actually, to evaluate the role of TG-lowering therapies among ESRD patients is very difficult through an observational study. The analyses would be biased with the confounding by indication. For example, those patients with hyperlipidemia required treatment of fibrate may originally have higher risks of CV events compared to those who do not need treatment, which must bias the results. Thus, this study was designed to compare not only the outcomes of patients using and not using fibrates but also those of patients using fibrates, statins, neither, and both. Studies have reported that among patients with ESRD, very low lipid profiles without requirements of statins or fibrates were conversely associated with higher risks of CV events, infections, and deaths (41, 42), which implies that these patients exhibited protein energy wasting (PEW), a complex of malnutrition and chronic inflammation, and had poor outcomes. If we simply compared the outcomes of fibrate users and non-users, patients with PEW would bias the results. On the other hand, although the direct effect of statins and fibrates are different (LDL-lowering vs.TG-lowering), physicians mostly prescribed these lipid-lowering agents in hopes of reducing risks of CV events. By comparing outcomes between fibrates-users and statins-users, we might, in some degree, reduce the confounding by indication. In this study, patients under treatment of fibrates not only had similar CV outcomes with those who did not receive lipid-lowering agents, but even exhibited slightly higher CV risks compared with patients under treatment of statins, which have been proved to exert less cardioprotective effect among ESRD patients (36, 37). Although an observational study is impossible to directly prove the cause and effect. These indirect evidences of this study implied that the treatment of fibrates may have no significant role in reducing CV events among patients with ESRD.

This study has main strength in being the only large-scale study focusing on the effects of fibrates on patients with ESRD, which enrolled more than 4,000 patients who used fibrates and employed a sufficient observation period. However, this study has some limitations should be acknowledged. First, although IPTW was used to adjust for possible confounding factors, some residual bias may have occurred due to the observational nature of the study. Second, some laboratory data, namely lipid profiles, blood sugar, glycosylated hemoglobin, and albumin, are not available in the NHIRD database, which made it difficult to balance the metabolic and nutritional profiles of the groups. Especially, a previous meta-analysis study enrolled patients with normal renal function has indicated that the fibrate effect on CV risks is greater in patients with higher TG levels (43). The lack of lipid profiles made it impossible to perform further subgroup analysis across different TG or cholesterol levels. Third, the dose of the lipid-lowering agents used by each patient was not available; thus, some heterogeneity in treatment may be inherent. Fourth, not all patients enrolled were new users of lipid-lowering medications. Therefore, the evaluation of possible side effects, which develop most commonly during the period soon after initiation, was out of the scope of this study.

## Conclusion

In contrast to our previous study involving patients with advanced CKD, which demonstrated that fibrates might delay the requirement of dialysis and reduce the risk of CV death among such patients, the present study focused on patients with ESRD and determined that the use of fibrates, even when combined with high-potency statins, is not associated with reduced all-cause mortality, CV deaths, and MACCEs among such patients. These results may inform the decisions of nephrologists regarding the treatment of hypertriglyceridemia in patients with ESRD and imply that prescribing fibrates for reducing CV risk in this population is unnecessary. This study was limited by its retrospective design and the lack of detailed lipid profiles. Additional randomized control trials and large-scale cohort studies with comprehensive laboratory data are warranted to verify our findings.

## Data availability statement

The original contributions presented in the study are included in the article/Supplementary material, further inquiries can be directed to the corresponding author/s.

## Ethics statement

This study was approved with a waiver of consent by the Institutional Review Board of Chang Gung Medical Foundation (Approval Number: 201900840B0).

## Author contributions

C-HC, C-LY, and H-HH contributed to conception and design of the study. C-YC, C-CL, Y-RT, W-YH, P-HC, and Y-CT collected and interpreted the data. C-CH and Y-RT analyzed the data. W-YH and C-LY wrote the manuscript. All authors contributed to manuscript revision, read, and approved the submitted version.

## Funding

This work was supported by grants from Chang Gung Memorial Hospital, Taiwan, (CMRPG5K0141). C-HC was supported by the Ministry of Science and Technology, Taiwan (109-2314-B-182A-124). The funder was not involved in the study design, collection, analysis, interpretation of data, the writing of this article or the decision to submit it for publication.

## Conflict of interest

The authors declare that the research was conducted in the absence of any commercial or financial relationships that could be construed as a potential conflict of interest.

## Publisher's note

All claims expressed in this article are solely those of the authors and do not necessarily represent those of their affiliated organizations, or those of the publisher, the editors and the reviewers. Any product that may be evaluated in this article, or claim that may be made by its manufacturer, is not guaranteed or endorsed by the publisher.
