# Supplementary material for: Use of fibrates is not associated with reduced risks of mortality or cardiovascular events among ESRD patients: A national cohort study
Source: Front Cardiovasc Med. 2022 Nov 9;9:907539. doi: 10.3389/fcvm.2022.907539 (PMC9681823; doi:10.3389/fcvm.2022.907539)
Supplement: Supplementary file 1 [file Table_1.DOCX]

**Supplemental Table 1.** Baseline characteristics of dialytic patients according to the use of fibrate and statin **after** IPTW

|  | After IPTW | | | | MASD |
| --- | --- | --- | --- | --- | --- |
| Variable | Non-user | Fibrate | Statin | Combination |  |
| Age, year | 64.5 ± 12.6 | 64.2 ± 12.1 | 64.4 ± 12.5 | 63.6 ± 11.9 | 0.08 |
| Age group |  |  |  |  | 0.09 |
| 20 – 64 years | 49.0 | 50.8 | 49.3 | 53.7 |  |
| 65 – 74 years | 28.6 | 28.7 | 28.9 | 26.9 |  |
| ≥ 75 years | 22.3 | 20.5 | 21.8 | 19.4 |  |
| Male | 48.3 | 48.4 | 48.2 | 45.9 | 0.06 |
| CKD duration, year | 5 [2, 8] | 5 [2, 8] | 5 [2, 8] | 5 [3, 8] | 0.03 |
| No. of outpatient visit in the previous year | 9 [1, 17] | 9 [1, 16] | 9 [2, 17] | 9 [1, 16] | 0.05 |
| Comorbid conditions |  |  |  |  |  |
| Hypertension | 90.3 | 90.6 | 90.4 | 89.8 | 0.03 |
| Diabetes mellitus | 74.1 | 75.1 | 74.5 | 74.7 | 0.02 |
| Atrial fibrillation | 3.5 | 3.5 | 3.4 | 2.7 | 0.05 |
| Liver cirrhosis | 3.2 | 2.6 | 2.9 | 1.5 | 0.09 |
| Peripheral artery disease | 4.8 | 4.5 | 4.5 | 4.3 | 0.02 |
| Dementia | 3.8 | 3.4 | 3.5 | 2.8 | 0.05 |
| Immune disease | 2.2 | 2.0 | 2.1 | 1.6 | 0.04 |
| History of event |  |  |  |  |  |
| Heart failure | 31.0 | 30.4 | 30.9 | 28.6 | 0.05 |
| Stroke | 24.3 | 23.5 | 23.8 | 22.9 | 0.03 |
| Myocardial infarction | 10.0 | 9.7 | 10.2 | 11.6 | 0.06 |
| Medication |  |  |  |  |  |
| ACEi / ARB | 49.5 | 51.4 | 50.7 | 53.7 | 0.09 |
| Beta blocker | 51.9 | 53.9 | 53.0 | 56.5 | 0.09 |
| DCCB | 69.9 | 70.6 | 71.0 | 69.2 | 0.04 |
| Loops diuretics | 59.9 | 61.1 | 61.2 | 62.6 | 0.06 |
| Spironolactone | 2.6 | 1.8 | 2.7 | 2.5 | 0.06 |
| NDCCB | 8.4 | 8.6 | 8.6 | 8.9 | 0.02 |
| Oral hypoglycemic agents | 41.6 | 43.6 | 42.7 | 45.0 | 0.07 |
| Insulin | 30.1 | 31.9 | 31.0 | 34.6 | 0.10 |
| Antiplatelet | 35.7 | 37.5 | 36.8 | 39.4 | 0.08 |
| Oral anticoagulants | 2.6 | 3.0 | 2.8 | 2.6 | 0.03 |
| NSAIDs | 15.0 | 15.2 | 15.0 | 14.9 | 0.01 |
| Steroid | 8.2 | 8.7 | 8.3 | 8.1 | 0.02 |
| Protom pump inhibitor | 17.0 | 18.2 | 17.1 | 18.6 | 0.04 |
| Ketosteril | 3.3 | 2.5 | 3.3 | 2.2 | 0.06 |
| Pentoxifylline | 13.7 | 14.1 | 14.1 | 14.0 | 0.01 |
| Sodium bicarbonate | 8.1 | 7.7 | 8.2 | 7.2 | 0.04 |
| Immunosuppressants | 1.4 | 1.1 | 1.5 | 1.7 | 0.05 |
| Vitamin D | 8.3 | 7.2 | 8.5 | 8.4 | 0.05 |
| Iron supplement | 15.4 | 14.7 | 15.7 | 14.7 | 0.03 |
| Calcium | 29.9 | 29.3 | 30.3 | 30.8 | 0.03 |
| Follow-up year | 3.3 ± 3.0 | 3.4 ± 3.0 | 3.4 ± 3.0 | 3.6 ± 3.1 | 0.04 |

Abbreviations: IPTW, inverse probability of treatment weighting; CKD, chronic kidney disease; MASD, maximum absolute standardized difference; ACEi, angiotensin converting enzyme inhibitor; ARB, angiotensin receptor blocker; DCCB, dihydropyrinde calcium channel blocker; NDCCB, non-dihydropyrinde calcium channel blocker; NSAIDs, non-steroidal anti-inflammatory drugs;

Data were presented as frequency (percentage), median [25^th^, 75^th^ percentile] or mean ± standard deviation.
