# Supplementary material for: Use of fibrates is not associated with reduced risks of mortality or cardiovascular events among ESRD patients: A national cohort study
Source: Front Cardiovasc Med. 2022 Nov 9;9:907539. doi: 10.3389/fcvm.2022.907539 (PMC9681823; doi:10.3389/fcvm.2022.907539)
Supplement: Supplementary file 2 [file Table_2.DOCX]

**Supplemental Table 2**. Baseline characteristics of dialytic patients according to the use of fibrate and moderate- to high-potency statins **before** IPTW

|  | Before IPTW | | | | MASD |
| --- | --- | --- | --- | --- | --- |
| Variable | Non-user  (*n* = 43,334) | Fibrate  (*n* = 3,027) | Moderate- to high-potency statins  (*n* = 19,508) | Combination  (*n* = 867) |  |
| Age, year | 65.7 ± 12.7 | 61.1 ± 12.3 | 62.7 ± 12.4 | 59.8 ± 11.1 | 0.47 |
| Age group |  |  |  |  | 0.48 |
| 20 – 64 years | 19,387 (44.7) | 1,824 (60.3) | 10,838 (55.6) | 595 (68.6) |  |
| 65 – 74 years | 12,742 (29.4) | 785 (25.9) | 5,337 (27.4) | 186 (21.5) |  |
| ≥ 75 years | 11,205 (25.9) | 418 (13.8) | 3,333 (17.1) | 86 (9.9) |  |
| Male | 21,171 (48.9) | 1,461 (48.3) | 9,269 (47.5) | 390 (45.0) | 0.08 |
| CKD duration, year | 5 [3, 8] | 4 [3, 7] | 5 [2, 8] | 5 [3, 8] | 0.13 |
| No. of outpatient visit in the previous year | 8 [1, 17] | 7 [1, 15] | 11 [3, 18] | 9 [2, 15] | 0.25 |
| Comorbid conditions |  |  |  |  |  |
| Hypertension | 38,856 (89.7) | 2,514 (83.1) | 18,258 (93.6) | 772 (89.0) | 0.36 |
| Diabetes mellitus | 30,744 (70.9) | 2,187 (72.2) | 15,925 (81.6) | 705 (81.3) | 0.24 |
| Atrial fibrillation | 1,697 (3.9) | 86 (2.8) | 596 (3.1) | 10 (1.2) | 0.15 |
| Liver cirrhosis | 1,562 (3.6) | 61 (2.0) | 474 (2.4) | 11 (1.3) | 0.13 |
| Peripheral artery disease | 2,066 (4.8) | 136 (4.5) | 921 (4.7) | 42 (4.8) | 0.02 |
| Dementia | 1,892 (4.4) | 78 (2.6) | 530 (2.7) | 21 (2.4) | 0.10 |
| Immune disease | 1,010 (2.3) | 59 (1.9) | 410 (2.1) | 19 (2.2) | 0.03 |
| History of event |  |  |  |  |  |
| Heart failure | 13,667 (31.5) | 724 (23.9) | 6,107 (31.3) | 229 (26.4) | 0.17 |
| Stroke | 10,813 (25.0) | 642 (21.2) | 4,598 (23.6) | 196 (22.6) | 0.09 |
| Myocardial infarction | 3,949 (9.1) | 227 (7.5) | 2,512 (12.9) | 105 (12.1) | 0.18 |
| Medication |  |  |  |  |  |
| ACEi / ARB | 19,573 (45.2) | 1,457 (48.1) | 11,520 (59.1) | 513 (59.2) | 0.28 |
| Beta blocker | 20,680 (47.7) | 1,602 (52.9) | 11,993 (61.5) | 554 (63.9) | 0.32 |
| DCCB | 28,856 (66.6) | 1,955 (64.6) | 15,278 (78.3) | 611 (70.5) | 0.08 |
| Loops diuretics | 24,119 (55.7) | 1,511 (49.9) | 13,953 (71.5) | 554 (63.9) | 0.30 |
| Spironolactone | 1,048 (2.4) | 50 (1.7) | 635 (3.3) | 24 (2.8) | 0.05 |
| NDCCB | 3,330 (7.7) | 255 (8.4) | 1,911 (9.8) | 85 (9.8) | 0.27 |
| Oral hypoglycemic agents | 16,209 (37.4) | 1,304 (43.1) | 9,893 (50.7) | 422 (48.7) | 0.34 |
| Insulin | 10,967 (25.3) | 1,121 (37.0) | 7,671 (39.3) | 449 (51.8) | 0.18 |
| Antiplatelet | 13,324 (30.7) | 1,109 (36.6) | 9,068 (46.5) | 409 (47.2) | 0.06 |
| Oral anticoagulants | 1,114 (2.6) | 90 (3.0) | 505 (2.6) | 29 (3.3) | 0.05 |
| NSAIDs | 6,456 (14.9) | 604 (20.0) | 2,661 (13.6) | 166 (19.1) | 0.58 |
| Steroid | 3,540 (8.2) | 222 (7.3) | 1,689 (8.7) | 60 (6.9) | 0.44 |
| Proton pump inhibitor | 7,240 (16.7) | 526 (17.4) | 3,470 (17.8) | 160 (18.5) | 0.13 |
| Ketosteril | 1,419 (3.3) | 50 (1.7) | 774 (4.0) | 21 (2.4) | 0.18 |
| Pentoxifylline | 5,190 (12.0) | 389 (12.9) | 3,548 (18.2) | 139 (16.0) | 0.16 |
| Sodium bicarbonate | 3,465 (8.0) | 157 (5.2) | 1,869 (9.6) | 60 (6.9) | 0.10 |
| Immunosuppressants | 600 (1.4) | 31 (1.0) | 326 (1.7) | 19 (2.2) | 0.10 |
| Vitamin D | 3,480 (8.0) | 241 (8.0) | 1,735 (8.9) | 80 (9.2) | 0.05 |
| Iron supplement | 6,352 (14.7) | 396 (13.1) | 3,417 (17.5) | 129 (14.9) | 0.12 |
| Calcium | 12,447 (28.7) | 961 (31.7) | 6,062 (31.1) | 274 (31.6) | 0.07 |
| Follow-up year | 3.2 ± 3.0 | 4.2 ± 3.5 | 3.1 ± 2.7 | 3.8 ± 3.3 | 0.63 |

Abbreviations: IPTW, inverse probability of treatment weighting; CKD, chronic kidney disease; MASD, maximum absolute standardized difference; ACEi, angiotensin converting enzyme inhibitor; ARB, angiotensin receptor blocker; DCCB, dihydropyrinde calcium channel blocker; NDCCB, non-dihydropyrinde calcium channel blocker; NSAIDs, non-steroidal anti-inflammatory drugs;

Data were presented as frequency (percentage), median [25^th^, 75^th^ percentile] or mean ± standard deviation.
