# Supplementary material for: Use of fibrates is not associated with reduced risks of mortality or cardiovascular events among ESRD patients: A national cohort study
Source: Front Cardiovasc Med. 2022 Nov 9;9:907539. doi: 10.3389/fcvm.2022.907539 (PMC9681823; doi:10.3389/fcvm.2022.907539)
Supplement: Supplementary file 3 [file Table_3.DOCX]

**Supplemental Table 3**. Baseline characteristics of dialytic patients according to the use of fibrate and moderate- to high-potency statins **after** IPTW

|  | After IPTW | | | | MASD |
| --- | --- | --- | --- | --- | --- |
| Variable | Non-user | Fibrate | Moderate- to high-potency statins | Combination |  |
| Age, year | 64.6 ± 12.6 | 64.2 ± 12.2 | 64.5 ± 12.5 | 63.6 ± 11.9 | 0.08 |
| Age group |  |  |  |  | 0.10 |
| 20 – 64 years | 48.7 | 50.7 | 49.0 | 53.6 |  |
| 65 – 74 years | 28.7 | 28.5 | 28.9 | 26.9 |  |
| ≥ 75 years | 22.6 | 20.8 | 22.1 | 19.5 |  |
| Male | 48.5 | 48.8 | 48.3 | 46.2 | 0.05 |
| CKD duration, year | 5 [2, 8] | 5 [2, 8] | 5 [3, 8] | 5 [3, 8] | 0.05 |
| No. of outpatient visit in the previous year | 9 [1, 17] | 9 [1, 16] | 9 [2, 17] | 9 [1, 16] | 0.05 |
| Comorbid conditions |  |  |  |  |  |
| Hypertension | 90.6 | 90.8 | 90.8 | 89.3 | 0.05 |
| Diabetes mellitus | 74.2 | 75.0 | 74.6 | 74.6 | 0.02 |
| Atrial fibrillation | 3.6 | 3.7 | 3.5 | 1.9 | 0.09 |
| Liver cirrhosis | 3.2 | 2.6 | 2.9 | 1.4 | 0.10 |
| Peripheral artery disease | 4.8 | 4.7 | 4.5 | 4.3 | 0.02 |
| Dementia | 3.8 | 3.4 | 3.6 | 3.3 | 0.03 |
| Immune disease | 2.2 | 2.0 | 2.1 | 1.9 | 0.03 |
| History of event |  |  |  |  |  |
| Heart failure | 31.2 | 30.3 | 31.1 | 28.7 | 0.05 |
| Stroke | 24.4 | 23.5 | 23.9 | 23.5 | 0.02 |
| Myocardial infarction | 10.1 | 9.7 | 10.4 | 12.1 | 0.08 |
| Medication |  |  |  |  |  |
| ACEi / ARB | 49.4 | 51.6 | 50.8 | 53.8 | 0.09 |
| Beta blocker | 52.1 | 54.4 | 53.4 | 56.7 | 0.09 |
| DCCB | 69.9 | 70.4 | 71.2 | 69.3 | 0.03 |
| Loops diuretics | 60.1 | 61.1 | 61.6 | 62.7 | 0.04 |
| Spironolactone | 2.7 | 1.8 | 2.8 | 2.4 | 0.03 |
| NDCCB | 8.3 | 8.7 | 8.5 | 9.1 | 0.08 |
| Oral hypoglycemic agents | 41.6 | 43.5 | 42.7 | 45.4 | 0.09 |
| Insulin | 30.1 | 32.0 | 31.0 | 34.3 | 0.02 |
| Antiplatelet | 35.7 | 37.6 | 37.0 | 40.2 | 0.04 |
| Oral anticoagulants | 2.6 | 3.1 | 2.8 | 2.7 | 0.06 |
| NSAIDs | 14.9 | 15.4 | 14.9 | 14.6 | 0.09 |
| Steroid | 8.2 | 8.9 | 8.5 | 7.8 | 0.05 |
| Proton pump inhibitor | 17.2 | 18.4 | 17.3 | 19.6 | 0.05 |
| Ketosteril | 3.4 | 2.6 | 3.4 | 2.7 | 0.02 |
| Pentoxifylline | 13.8 | 14.4 | 14.3 | 13.8 | 0.04 |
| Sodium bicarbonate | 8.3 | 7.9 | 8.6 | 7.6 | 0.03 |
| Immunosuppressants | 1.4 | 1.2 | 1.5 | 1.4 | 0.06 |
| Vitamin D | 8.3 | 7.3 | 8.5 | 7.9 | 0.04 |
| Iron supplement | 15.4 | 14.7 | 15.6 | 15.3 | 0.03 |
| Calcium | 29.5 | 29.0 | 30.1 | 30.4 | 0.03 |
| Follow-up year | 3.2 ± 3.0 | 3.4 ± 3.0 | 3.4 ± 3.0 | 3.5 ± 3.1 | 0.04 |

Abbreviations: IPTW, inverse probability of treatment weighting; CKD, chronic kidney disease; MASD, maximum absolute standardized difference; ACEi, angiotensin converting enzyme inhibitor; ARB, angiotensin receptor blocker; DCCB, dihydropyrinde calcium channel blocker; NDCCB, non-dihydropyrinde calcium channel blocker; NSAIDs, non-steroidal anti-inflammatory drugs;

Data were presented as frequency (percentage), median [25^th^, 75^th^ percentile] or mean ± standard deviation.
