# Supplementary material for: Use of fibrates is not associated with reduced risks of mortality or cardiovascular events among ESRD patients: A national cohort study
Source: Front Cardiovasc Med. 2022 Nov 9;9:907539. doi: 10.3389/fcvm.2022.907539 (PMC9681823; doi:10.3389/fcvm.2022.907539)
Supplement: Supplementary file 4 [file Table_4.DOCX]

**Supplemental Table 4.** Follow up outcome for the dialytic patients according to the long-term use of fibrate and statin

|  | Incidence$ |  | HR (95% CI) (Column vs. Row) | | |
| --- | --- | --- | --- | --- | --- |
| Outcome / group | (95% CI) |  | Fibrate | Statin | Combination |
| All-cause mortality |  |  |  |  |  |
| Non-user | 17.5 (17.3–17.8) |  | 0.98 (0.92–1.05) | **0.92 (0.90–0.95)*** | 1.03 (0.92–1.14) |
| Fibrate | 13.4 (12.6–14.2) |  | - | 0.94 (0.88–1.005) | 1.05 (0.93–1.18) |
| Statin | 14.2 (13.9–14.5) |  | - | - | 1.11 (0.998–1.24) |
| Combination | 13.1 (11.7–14.5) |  | - | - | - |
| Cardiovascular death |  |  |  |  |  |
| Non-user | 9.1 (9.0–9.3) |  | 0.99 (0.90–1.08) | **0.94 (0.91–0.97)*** | 1.07 (0.93–1.24) |
| Fibrate | 6.8 (6.2–7.4) |  | - | 0.95 (0.87–1.04) | 1.09 (0.92–1.28) |
| Statin | 7.7 (7.5–7.9) |  | - | - | 1.14 (0.99–1.32) |
| Combination | 7.0 (6.0–8.0) |  | - | - | - |
| MACCE# |  |  |  |  |  |
| Non-user | 12.7 (12.5–12.9) |  | 1.00 (0.92–1.08) | **0.95 (0.93–0.98)*** | 1.12 (0.99–1.27) |
| Fibrate | 9.9 (9.2–10.7) |  | - | 0.96 (0.89–1.04) | 1.13 (0.98–1.30) |
| Statin | 11.6 (11.3–11.9) |  | - | - | **1.17 (1.04–1.33)** |
| Combination | 11.0 (9.7–12.4) |  | - | - | - |

Abbreviations: HR, hazard ratio; CI, confidence interval; MACCE, major adverse cardiac and cerebrovascular events;

$ Incidence density was presented as event numbers per 100 person-years;

# Composite of cardiovascular death, ischemic stroke, or acute myocardial infarction;

* *P* < 0.05.
